# Supplementary material for: Evaluating Effects of AIV Infection Status on Ducks Using a Flow Cytometry-Based Differential Blood Count
Source: Microbiol Spectr. 2023 Jun 15;11(4):e04351-22. doi: 10.1128/spectrum.04351-22 (PMC10434237; doi:10.1128/spectrum.04351-22)
Supplement: Supplemental file 1 — Supplemental material. Download spectrum.04351-22-s0001.pdf, PDF file, 1.0 MB [file spectrum.04351-22-s0001.pdf]

Supplementary Information for

Evaluating effects of AIV infection status on ducks using a flow cytometry based differential blood count

Elinor Jax[Δ*], Elena Werner[Δ], Inge Müller, Beatrice Schaerer, Marina Kohn, Jenny Olofsson, Jonas Waldenström, Robert Kraus, Sonja Härtle[*]

Δ these two authors contributed equally

Elinor Jax[*]

Email: ejax@ab.mpg.de

Sonja Härtle[*]

Email: Sonja.Haertle@lmu.de

This file includes:

Supplementary figures S1 to S8

Supplementary tables S1 to S4

Supplementary information

Supplementary figures

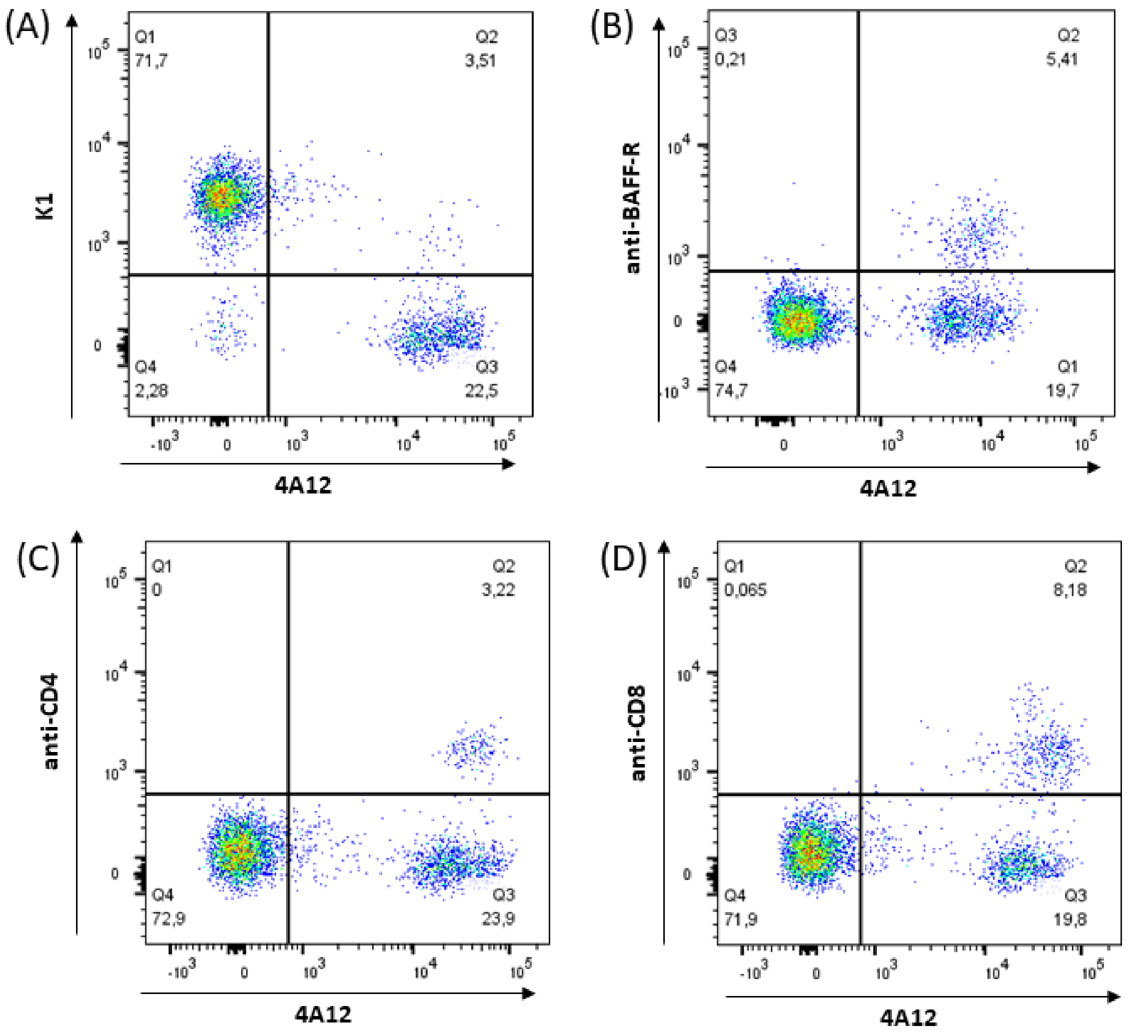

Figure S1 4A12

Leukocytes were isolated from blood by density gradient centrifugation and stained with viability dye (Fixable Viability dye eFluor™ 780), mab 4A12 and markers for thrombocytes (A), B cells (B), CD4plus cells (C) and CD8plus cells (D) followed by the respective fluorochrome conjugated secondary antibodies (IgG1-APC, IgG2a-FITC, IgG2b-FITC). For anti-BAFF-R staining, a 2C4-APC direct conjugate was used. Plots are gated for viable leukocytes.

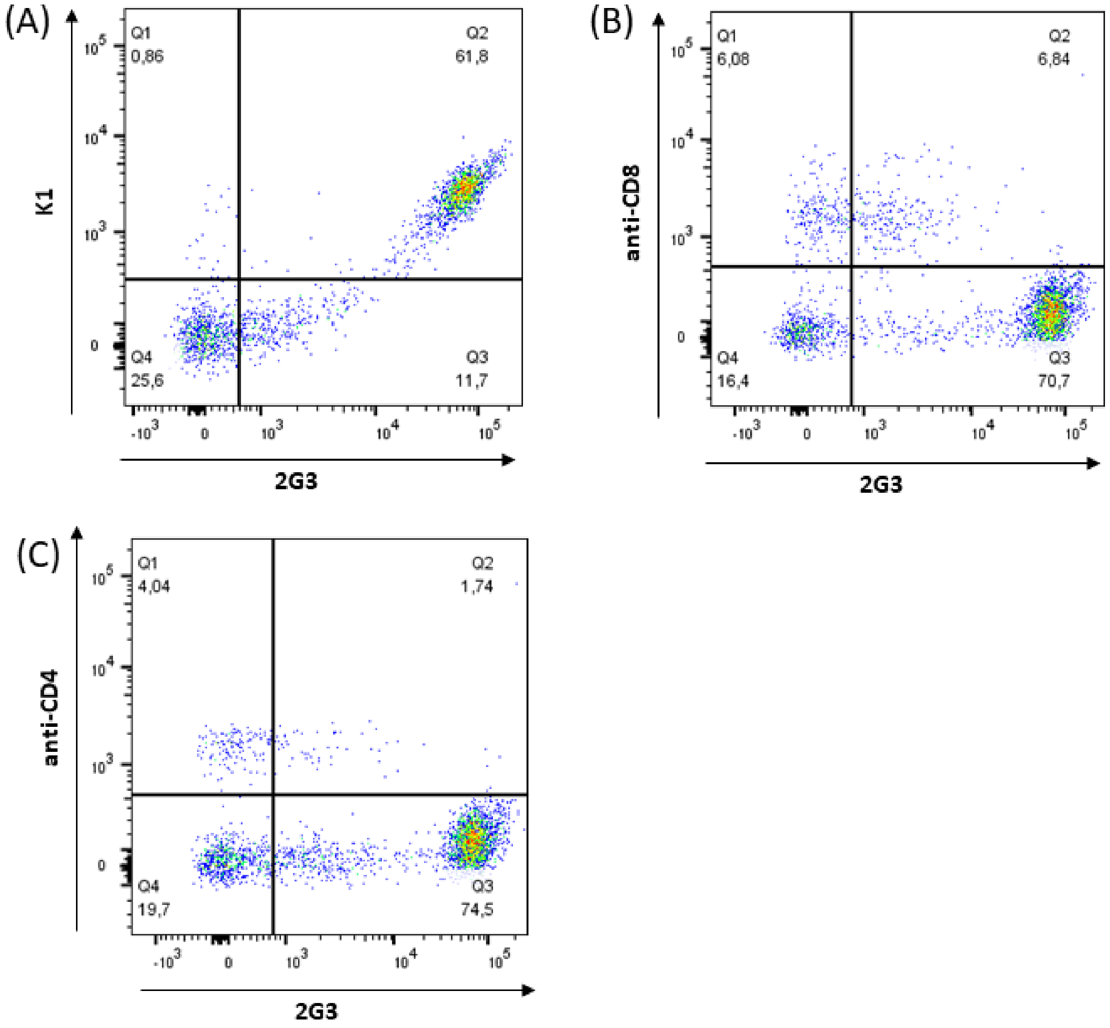

Figure S2 2G3

Leukocytes were isolated from blood by density gradient centrifugation and stained with a viability dye (Fixable Viability dye eFluor™ 780), mab 2G3 and markers for thrombocytes (A), CD8plus cells (B) and CD4plus cells (C) followed by the respective fluorochrome conjugated secondary antibodies (IgG1-APC, IgG2a-FITC, IgG2b-FITC). Plots are gated for viable leukocytes.

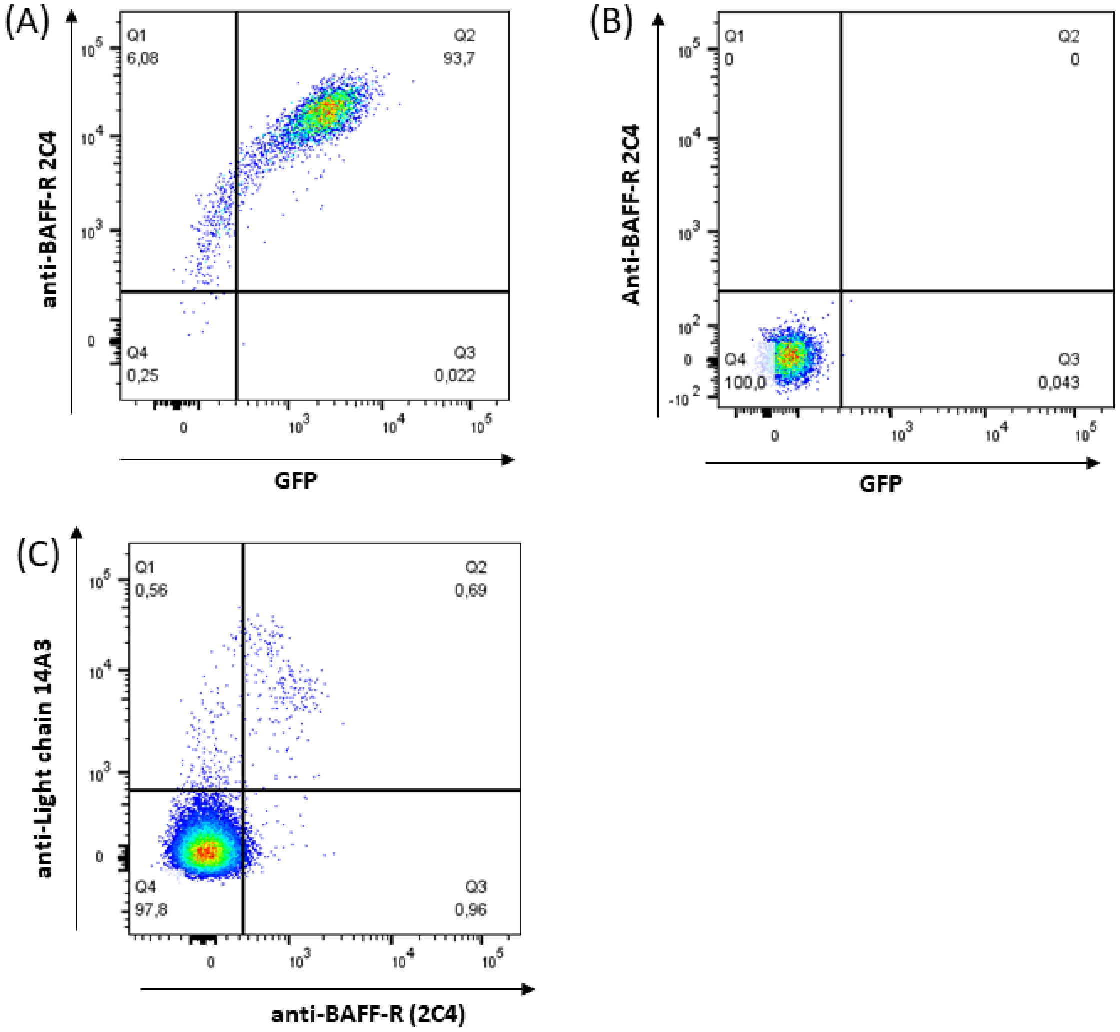

Figure S3 2C4

HEK293 cells, stably transfected with pcDNA™3.1- BAFF-R-GFP (A) or untransfected controls (B) were stained with mab 2C4 to demonstrate antibody specificity. Leukocytes were isolated from blood by density gradient centrifugation and stained with viability dye (Fixable Viability dye eFluor™ 780), mab 14A3 detecting the immunoglobulin light chain and anti-chicken-BAFF-R antibody 2C4 (C). Plots are gated for viable, single cells.

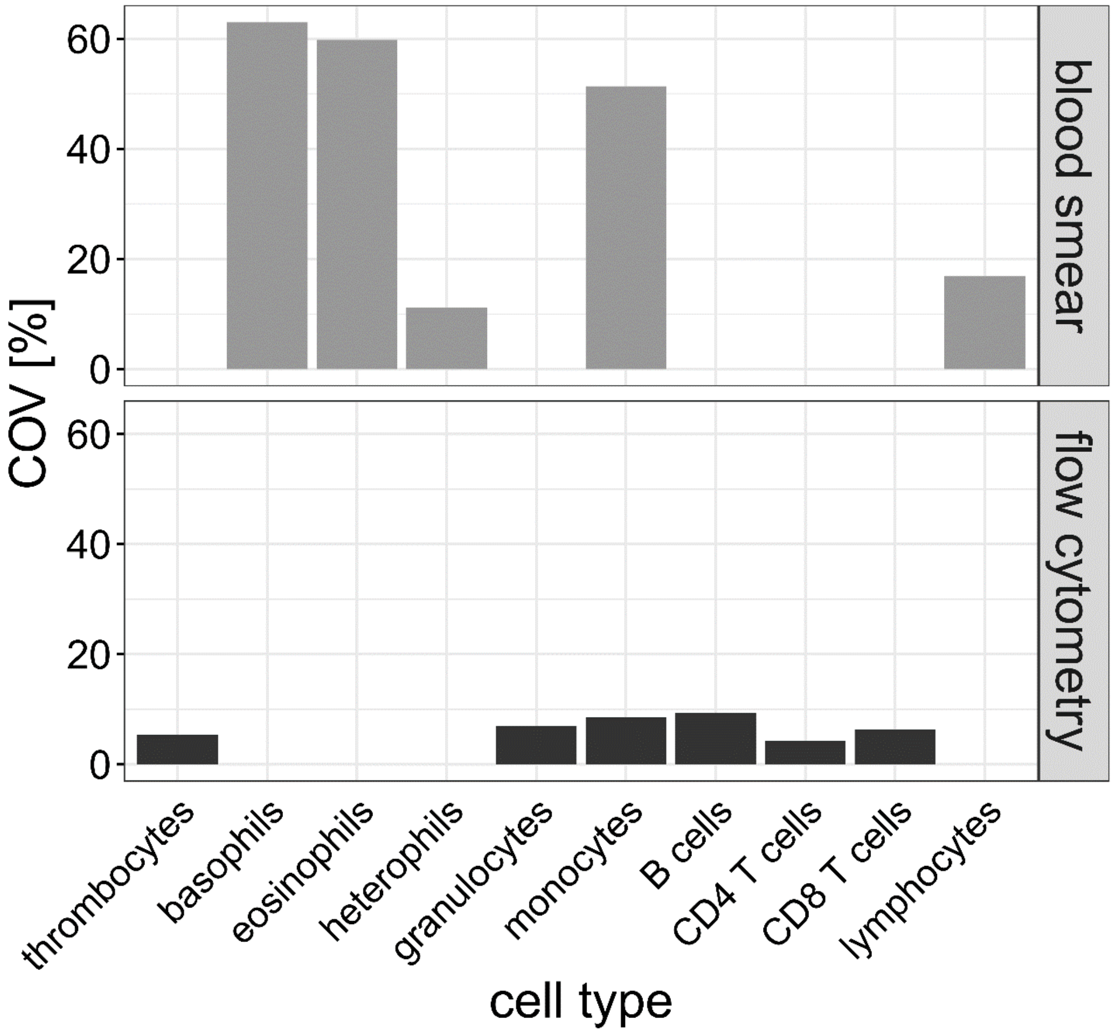

Figure S4. Precision of flow cytometric and microscopic WBC differential

Five aliquots from one EDTA-blood sample were either analysed by flow cytometry or used to prepare blood smears, which were subjected to a Wright–Giemsa staining and analysed by microscope-based cell counting according to the modified Campbell method. Shown are the coefficient of variation (COV) in percentage for each cell population. Cell populations that cannot be estimated for either method do not have a bar.

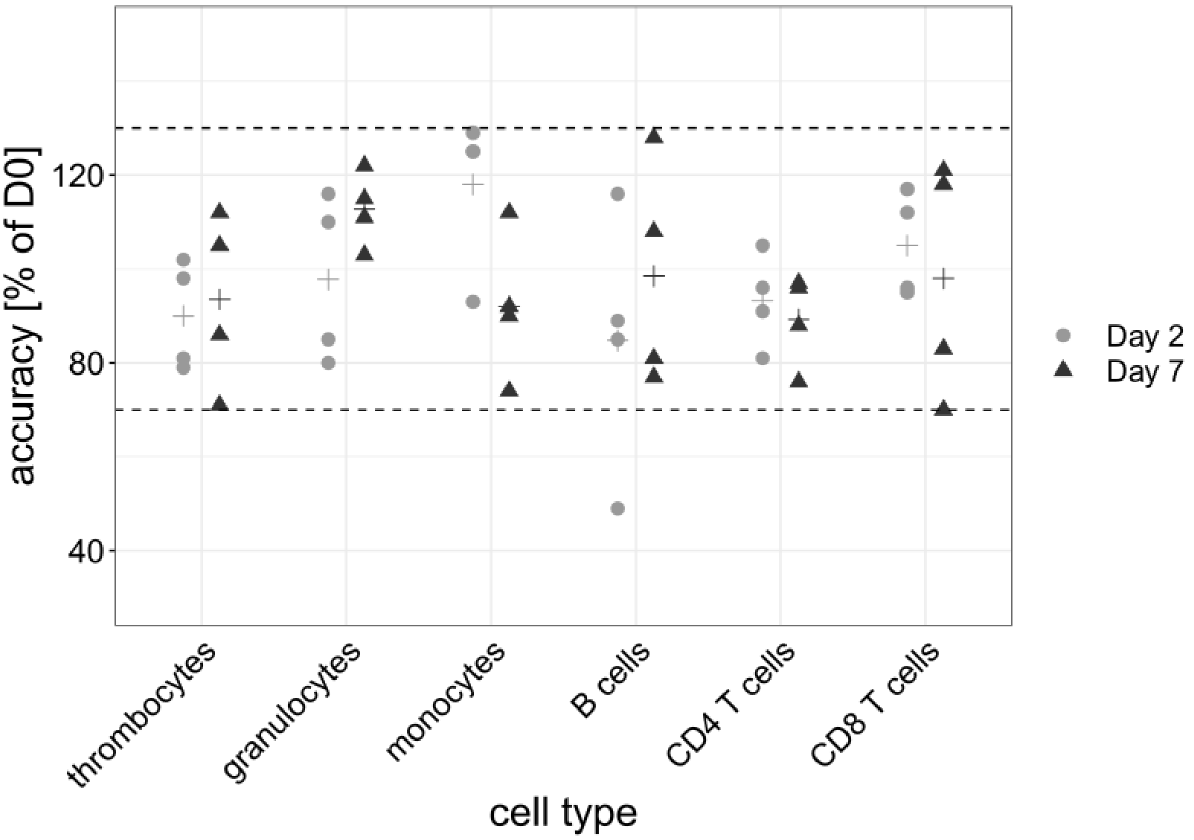

40

41 ## Figure S5. Effect of sample fixation

To determine whether stabilisation of blood samples with transfix reagent does alter measurements, EDTA blood samples from four ducks were split in three technical replicates and each of them was analysed by flow cytometry as a fresh sample a few hours post blood withdrawal or treated with Transfix® reagent and analysed as stabilised samples two and seven days later The coefficient of variation (COV) for all technical triplicates, beside a single granulocyte count, was below 20%, the vast majority of samples had a COV below 10%. Accuracy of D2 (grey circle) and D7 (black triangle) measurements compared to D0 counts was determined for every cell population according to the formula: mean of triplicate (D0) / mean of triplicate (D2 or D7) * 100. Each symbol in the graph represents the mean of a technical triplicate, + represent the mean for a cell population. Black dashed lines mark the borders of acceptance (100±30%).

42

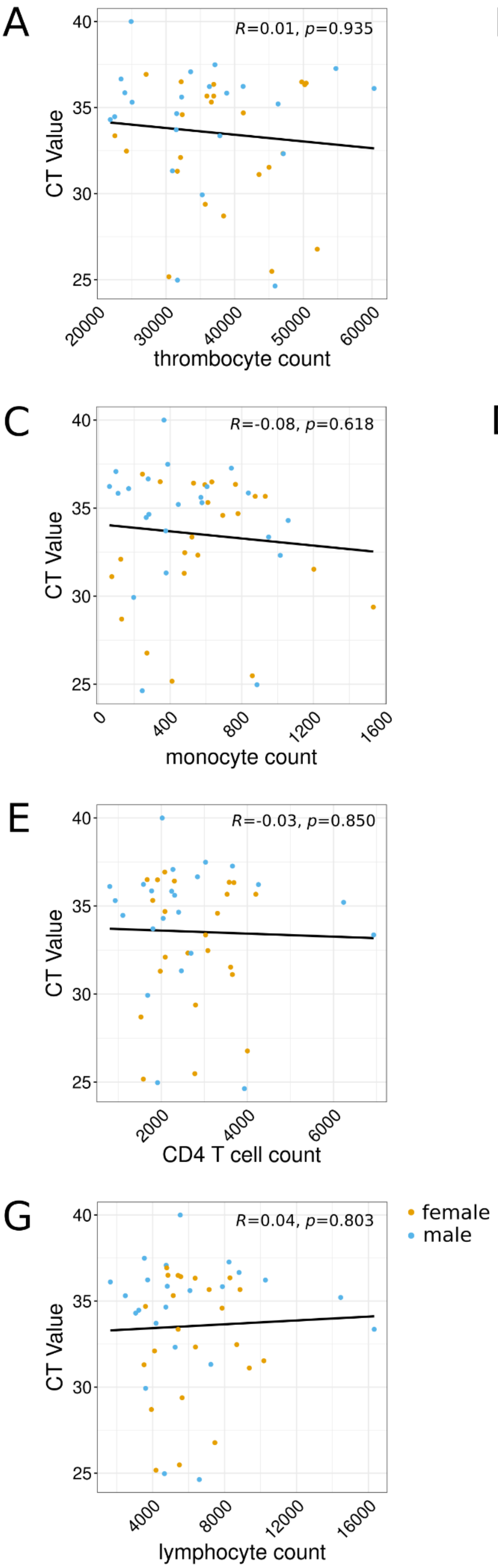
A
R=0.01, p=0.935
CT Value
thrombocyte count
B
R=-0.22, p=0.144
CT Value
granulocyte count
C
R=-0.08, p=0.618
CT Value
monocyte count
D
R=-0.00, p=0.981
CT Value
B cell count
E
R=-0.03, p=0.850
CT Value
CD4 T cell count
F
R=0.11, p=0.472
CT Value
CD8 T cell count
G
R=0.04, p=0.803
CT Value
lymphocyte count
female
male

Figure S6. Relationship between CT value and white blood cell counts

Correlation plots showing the relationship between the viral RT-PCR cycle threshold (CT) value and number of (A) thrombocytes, (B) granulocytes, (C) monocytes, (D) B cells, (E) CD4 T cells, (F) CD8 T cells, and (G) lymphocytes in all AIV positive juvenile ducks. The Spearman's rank correlation coefficient (*R*) and the *p*-value (*p*) for each white blood cell type are displayed. The CT value gives an approximate indication of how much viral genetic material is in a sample, with lower CT values indicating a higher concentration of viral genetic material.

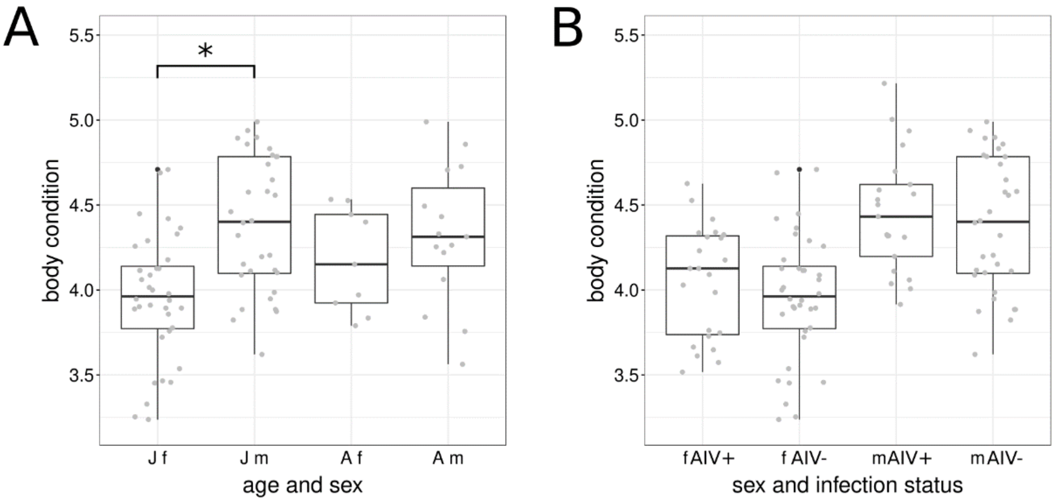

Figure S7. Body condition in mallards of different sex, age and AIV infection status

The body condition (body weight/wing length) are presented as boxplots for A) mallards of different age and sex and B) juvenile mallards of different sex and infection status. The horizontal line displays the median, the box includes the second and the third quantile, the whiskers include all values within the 1.5 interquartile range, black dots represent outliers. The body condition for individual birds is shown as grey dots. J = juvenile, A = adult, f = female, m = male, AIV+ = avian influenza virus positive, AIV- = avian influenza virus negative. Groups with a significant difference ($p < 0.05$) in the mean are reported with an asterisk in the figure.

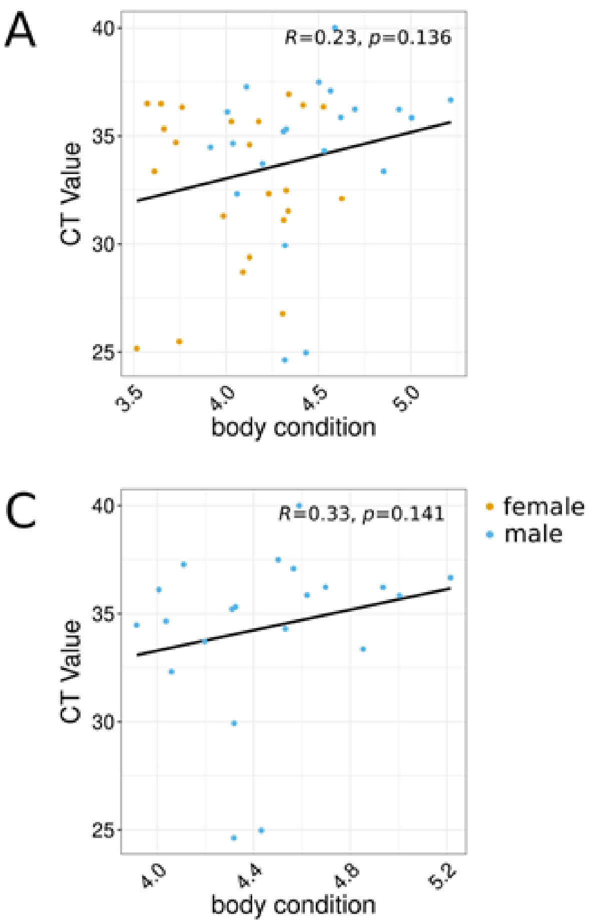

Figure S8. Relationship between CT value and body condition

Correlation plots showing the relationship between the CT value and the body condition in all A) AIV positive juveniles, B) AIV positive juvenile females, C) AIV positive juvenile males. The Spearman's rank correlation coefficient (*R*) and the p-value (*p*) for each test are displayed. The CT value gives an approximate indication of how much viral genetic material is in a sample, with lower Ct values indicating a higher concentration of viral genetic material. The body condition was estimated by dividing the body weight with the wing length for each individual bird.

Supplementary tables

Table S1 Observed blood cell count values for AIV negative mallards, grouped according to age and sex.

The values show the observed blood cell population counts in cells/µL whole blood. Given are the range and the mean +/- SD for each group and the respective cell population. Blood cell values are based on the observed cell counts of n = 116 AIV negative mallards, including n = 41 juvenile males (Jm), n = 44 juvenile females (Jf), n = 12 adult females (Af) and n = 19 adult males (Am).

**Cell type**		**Jf**	**Jm**	**Af**	**Am**
**Thrombocytes**	mean +/- SD	37860+/-11483	40066+/-9175	38998+/-7907	39145+/-10706
	range	21280-65319	25990-62886	25934-54384	21358-66748
**Granulocytes**	mean +/- SD	8488+/-3999	8696+/-4619	8620+/-3230	7589+/-2639
	range	2246-21003	3414-28186	4832-16226	3731-12474
**Monocytes**	mean +/- SD	617+/-487	571+/-326	534+/-277	359+/-245
	range	123-2864	203-1176	101-875	13-878
**B cells**	mean +/- SD	895+/-642	1222+/-807	560+/-338	499+/-468
	range	150-2667	267-3865	122-1120	116-1785
**CD8 T cells**	mean +/- SD	2595+/-1324	3012+/-1602	2052+/-1119	1791+/-1151
	range	704-7108	1038-8385	922-4551	382-5510
**CD4 T cells**	mean +/- SD	2304+/-830	3032+/-1179	1747+/-612	1672+/-824
	range	919-4982	1245-7263	863-3097	469-3943
**Lymphocytes**	mean +/- SD	5795+/-2415	7266+/-3067	4359+/-1962	3962+/-2158
	range	2289-14757	3606-16433	2109-8741	1559-9967

Table S2 Estimated blood cell count values for AIV negative mallards, grouped according to age and sex

Given are the estimated mean count in cells/µL and the respective 95 % credible interval (CrI) for each group and cell population. The count values were estimated using a normal linear model for age, sex and its interaction as fixed effects. The model was run, based on the observed blood cell counts of n = 116 AIV negative mallards, including n = 41 juvenile males (Jm), n = 44 juvenile females (Jf), n = 12 adult females (Af) and n = 19 adult males (Am).

Cell type		Jf	Jm	Af	Am
**Thrombocytes**	estimated mean	37860	40066	38998	39145
	95 % CrI	34782-40880	36890-43225	33183-45038	34370-43815
**Granulocytes**	estimated mean	8488	8696	8620	7589
	95 % CrI	7268-9673	7451-9943	6316-10917	5778-9416
**Monocytes**	estimated mean	617	571	534	359
	95 % CrI	504-734	450-689	319-750	187-534
**B cells**	estimated mean	895	1222	560	499
	95 % CrI	702-1092	1017-1423	182-934	198-802
**CD8 T cells**	estimated mean	2595	3012	2052	1791
	95 % CrI	2178-3007	2581-3441	1263-2874	1150-2418
**CD4 T cells**	estimated mean	2304	3032	1747	1672
	95 % CrI	2019-2589	2742-3335	1217-2294	1228-2110
**Lymphocytes**	estimated mean	5795	7266	4359	3962
	95 % CrI	4993-6578	6471-8065	2857-5840	2780-5147

Table S3 Observed blood cell count values for AIV positive and AIV negative mallards, grouped according to sex

The values show the observed blood cell population counts in cells/µL whole blood. Given are the range and the mean +/- SD for each group and the respective cell population. Blood cell values were obtained from n = 131 juvenile mallards of both sexes, which were tested to be AIV positive or AIV negative (n = 23 AIV positive males (mAIV+), n = 23 AIV positive females (fAIV+), n = 41 AIV negative males (mAIV-), n = 44 AIV negative females (fAIV-)).

**Cell type**		**f AIV+**	**f AIV-**	**m AIV+**	**m AIV-**
**Thrombocytes**	mean +/- SD	38167+/-8592	37860+/-11483	35407+/-10291	40066+/-9175
	range	22560-52028	21280-65319	21885-60305	25990-62886
**Granulocytes**	mean +/- SD	9675+/-4365	8488+/-3999	9504+/-3845	8696+/-4619
	range	4053-23352	2246-21003	3060-18901	3414-28186
**Monocytes**	mean +/- SD	600+/-342	617+/-487	474+/-309	571+/-326
	range	75-1534	123-2864	62-1059	203-1176
**B cells**	mean +/- SD	963+/-574	895+/-642	1004+/-847	1222+/-807
	range	171-2062	150-2667	79-3254	267-3865
**CD8 T cells**	mean +/- SD	2423+/-1096	2595+/-1324	2481+/-1619	3012+/-1602
	range	667-5055	704-7108	433-6136	1038-8385
**CD4 T cells**	mean +/- SD	2697+/-891	2304+/-830	2649+/-1518	3032+/-1179
	range	1217-4196	919-4982	804-6932	1245-7263
**Lymphocytes**	mean +/- SD	6084+/-2085	5795+/-2415	6135+/-3626	7266+/-3067
	range	2435-10184	2289-14757	1648-16322	3606-16433

Table S4 Estimated blood cell count values for AIV positive and AIV negative mallards, grouped according to sex

Listed is the estimated mean count in cells/µL the respective the 95 % credible interval (CrI) for each group and cell population are listed. The estimated lymphocyte count includes all B- and T cells. The count values were estimated using a normal linear model for AIV-infection, sex and their interaction as fixed effects. The model was run, based on the observed blood cell counts of n = 131 juvenile mallards of both sexes, which were tested to be AIV positive or AIV negative (n = 23 AIV positive males (mAIV+), n = 23 AIV positive females (fAIV+), n = 41 AIV negative males (mAIV-), n = 44 AIV negative females (fAIV-)).

**Cell type**		**fAIV+**	**fAIV-**	**mAIV+**	**mAIV-**
**Thrombocytes**	estimated mean	38167	37860	35407	40066
	95 % CrI	34005-42378	34818-40852	31253-39543	36938-43253
**Granulocytes**	estimated mean	9675	8488	9504	8696
	95 % CrI	7934-11435	7235-9740	7759-11266	7360-10019
**Monocytes**	estimated mean	600	617	474	571
	95 % CrI	444-759	501-732	312-632	451-692
**B cells**	estimated mean	963	895	1004	1222
	95 % CrI	660-1259	676-1110	701-1308	995-1446
**CD8 T cells**	estimated mean	2423	2595	2481	3012
	95 % CrI	1836-3016	2167-3037	1893-3079	2578-3461
**CD4 T cells**	estimated mean	2697	2304	2649	3032
	95 % CrI	2255-3159	1972-2633	2196-3111	2691-3367
**Lymphocytes**	estimated mean	6084	5795	6135	7266
	95 % CrI	4919-7243	4968-6650	4975-7296	6383-8151
